# Clinical and laboratory profile of children with Cystic Fibrosis: Experience of a tertiary care center in Pakistan

**DOI:** 10.12669/pjms.333.12188

**Published:** 2017

**Authors:** Danish Abdul Aziz, Abdul Gaffar Billoo, Ahad Qureshi, Misha Khalid, Salman Kirmani

**Affiliations:** 1 Dr. Danish Abdul Aziz. MBBS, MRCPCH, FCPS. Senior Instructor, Department of Paediatrics, Aga Khan University Hospital, Karachi, Pakistan; 2 Prof. Dr. Abdul Gaffar Billoo, MBBS, MRCP, FRCP. Department of Paediatrics, Aga Khan University Hospital, Karachi, Pakistan; 3 Dr. Ahad Qureshi, MBBS. Graduate from Aga Khan University Hospital, Karachi, Pakistan. Aga Khan University Hospital, Karachi, Pakistan; 4 Misha Khalid, Fourth Year Medical Student, Aga Khan University Hospital, Karachi, Pakistan; 5 Dr. Salman Kirmani, MBBS, FAAP. Associate Professor, Department of Paediatrics, Aga Khan University Hospital, Karachi, Pakistan

**Keywords:** Cystic Fibrosis, Delta F508, Genetic mutations analysis, Sweat chloride test

## Abstract

**Objective:**

To determine the clinical presentation, diagnostic investigations and laboratory workup done in admitted children with cystic fibrosis at Aga Khan University Hospital Karachi, Pakistan.

**Methods:**

This is athree years retrospective study from January 2013 to December 2015 conducted at The Aga Khan University Hospital Karachi Pakistan, enrolling admitted patient from birth to 15 years of either gender, diagnosed with CF on the basis of clinical features and positive sweat chloride test. Different clinical presentations were noted including initial presentations. Sweat chloride values more than 60mmol/L were labeled as positive and consistent with diagnosis of CF. Available Delta F-508 mutation analyses were noted. Relevant laboratory and radiological investigations including sputum culture and HR-CT chest findings were documented. Results were analyzed using SPSS version 20.

**Results:**

Total 43 children were selected according to the inclusion criteria. Chronic cough (69.76%) was the most common initial clinical presentation. Mean age at onset of symptoms was 14.41± 26.18 months and mean age at diagnosis was 47.20 ± 45.80 months Respiratory features were most common in our cohort including chronic productive cough (90.71%), recurrent bronchopneumonia (72.09%) and asthma like presentation (44.19%) with wheezing and cough. 86% patients presented with failure to thrive. Gastroenterological features including steatorrhea were seen in 55.81% patients and 44.19% patients had abdominal distension. Mean sweat chloride value in our population was 82.70± 22.74. Gene analysis for Delta F-508 was identified in 12 (27.90%) patients. Bronchiectatic pulmonary changes on HRCT were seen in 18 patients (41.86%). Pseudomonas grew in 12 patients (27.90%) in sputum cultures at the time of diagnosis.

**Conclusion:**

Respiratory presentations predominate in CF children followed by gastrointestinal features. Nearly half of our patient had bronchiectatic changes on CT scan chest and more than quarter had pseudomonas colonization in the airways at the time of diagnosis. Delta F-508 mutation was found to be uncommon in our study population. There is significant delay in diagnosing patients with CF.

## INTRODUCTION

Cystic fibrosis (CF) is life limiting genetic disorder common in Caucasians of North America, Australia and Europe.^1^,^2^ Mutated CF transmembrane conductance regulator (CFTR) epithelial chloride channel desiccates secretions in respiratory airways, hepatobiliary-pancreatic ducts and in other tissue linings, resulting in gradual and persistent organ damage.^3^ Estimated gene frequency of cystic fibrosis varies in different ethnic groups with highest incidence in Caucasians (1 in 2,500). Approximate incidence of CF in South Asians immigrants settled in UK is about 1:10,000 to 1:12,000.^4^ CF is one of the under diagnosed disease in Pakistan.^5^ Case identification on basis of history, examination and relevant laboratory investigations are fundamental for successful diagnosis.

Patients with CF usually present in the first two years of life with chronic productive cough, recurrent pneumonia, resistant asthma, failure to thrive, chronic diarrhea (steattorhea) and dehydration. Neonatal Screening program measuring activated serum trypsinogen level has been conducted in developed countries to pick the potential cases early in life. In underdeveloped countries, screening programs are not available and most of the cases are diagnosed on the basis of clinical presentation and supported by sweat chloride test and genetic analysis. Genetic diagnosis in CF is crucial in defining the disease behavior and prognosis. Most commonly reported CF related genetic mutation among Caucasians is Delta F-508.^6^ Limited range of genetic mutation analysis is available in our part of world. Mutations data reported in few studies in Pakistani CF suggested that D-F508 mutation is rare in this population.^5^ This is in contrast to some studies which showed predominance of this mutation in Pakistani communitywith CF.^7^,^8^

Aim of this study was to highlightthe main clinical features seen in CF children in our population and pertinent laboratory workup done to diagnose and manage these patients.

## METHODS

A three years retrospective study conducted at Aga Khan University Hospital from January 2013 to December 2015 after approval of Ethical review committee of the university. Admitted patients diagnosed with Cystic fibrosis on the basis of their clinical features and positive sweat chloride test from birth to 15 years of either gender were enrolled in this study. Patients were categorized in five groups for the age at the onset of symptoms and age at diagnosis. These CF patients were studied further for their initial clinical signs or symptoms. Sweat chloride values were noted for their mean value and standard deviation. Sweat was stimulated by means of pilocarpine iontophoresis and sweat collection done by Wescor macroduct sweat collection system as adopted by chemical pathology laboratory of the hospital. Patients with sweat chloride values more than 60mmol/L were labeled as positive and consistent with diagnosis of CF. Around a decade ago, Delta F-508 mutation analysis was started at Aga Khan University Hospital molecular laboratory on the basis of reported genetic mutations in CF Children from our adjoining region.^7^,^8^ All patients with positive sweat chloride were analyzed for Delta F-508 genetic mutation. Patients with positive Delta F-508 gene mutation status were further categorized in homozygous and heterozygous. Family history or history of siblings with CF was noted along with parents who have consanguineous relations. General body parameters were noted along with common physical examination findings. Neonatal, respiratory, gastrointestinal, cardiovascular and other presentations were documented for these children.

Complete blood count, Electrolytes, stool for fat globules, Blood culture, and X rays findings were noted. High resolution Computed Tomography scan (HRCT) of chest done at the time of diagnosis were studied and categorized into six different categories according to radiological findings. Sputum for organism growth was studied further to find out first organism to colonize the airway at the time of diagnosis.

Results were analyzed using SPSS version 20. Data was summarized using mean, standard deviation, numbers and percentages for different variables.

## RESULTS

**Fig.1:**
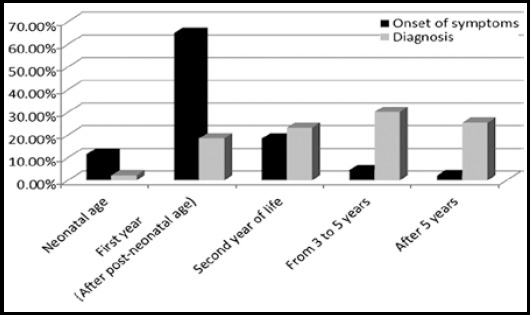
Comparison of age of onset of symptoms and diagnosis.

**Fig.2:**
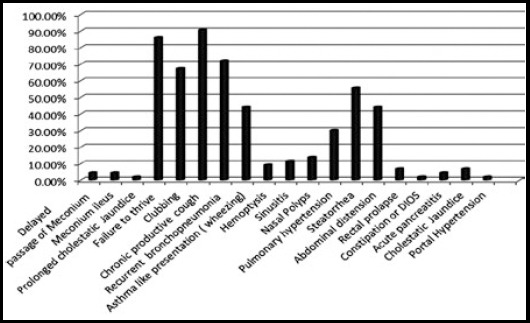
Clinical Presentations of CF children (DIOS=Distal intestinal obstruction syndrome).

This study enrolled 43 children with clinical features suggestive of Cystic fibrosis and with positive sweat chloride test. Mean age in our population was 56.00 ± 33.71 months (4.6 years). Male to female ratio was 1.3:1. Different demographic feature of our population are shown in Table-I. Family history was positive in four patients (9.30%) and 24 (55.81%) children have parents with consanguinity. Comparison of different age groups for onset of symptoms and diagnosis is shown in Fig-1.. Different diagnostic test done in our patients are presented in Table-II. Chronic cough was the most common initial clinical presentation in our experience. (Table-III), Clinical presentations noted in our cohort including neonatal, respiratory gastrointestinal and hepatobiliary systems are presented in Fig-2.. Results of different investigations done on CF patients are shown in Table-IV.

**Table-I:** Demographic Features and General Parameters of CF Children (N=43).

*Age in months*	*Mean: 56.00 months, Std. Deviation: ± 33.71, Range 1 month -178 months*
Male	25 (58.13%)
Female	18 (41.86%)
Male to female Ratio	1.3 : 1
Mean age at onset of symptoms	14.41± 26.18 months
Mean age at diagnosis	47.20 ± 45.80 months
Height below 3rd centile (short stature)	24 (55.81%)
Weight below 3rd centile	34 (79.06%)

**Table-II:** Diagnostic Tests in CF children.

*Sweat chloride test results*	*Mean Value: 82.70, Std. Deviation: ± 22.74*
Gene analyses for Delta F-508	12 (27.90%)
Negative for Delta F-508	31 (72.09%)
Heterozygous	5 (11.62%)
Homozygous	7 (16.27%)

**Table-III:** Initial clinical presentations (N=43) of study participants.

Delayed passage of Meconium or Meconium ileus	4 (9.30%)
Prolonged Cholestatic Jaundice	1 (2.32%)
Chronic cough	30 (69.76%)
Steatorrhea	5 (11.62%)
Failure to thrive only	1 (2.32%)
Pseudo barter syndrome with electrolyte imbalance	2 (4.65%)

**Table-IV:** Laboratory and Radiological Investigations (N=43) in CF patients.

*Complete blood count*	*Mean values with Standard deviation*	*Electrolytes*	*Mean values with Standard deviation*
Hemoglobin (g/L)	8.67 ± 3.42	Sodium (mEq/L)	135.23 ± 2.23
White blood count (X 10E9/L)	13.23 ± 4.89	Potassium (mEq/L)	3.39 ± 1.57
Platelets (X 10E9/L)	265.31 ±127.16	Chloride (mEq/L)	95.21 ± 3.81
C-reactive protein (mg/dl)	6.21 ±5.65	Bicarbonate (mEq/L)	22.45 ± 2.25

Blood Culture	N=43 (%)	Sputum / Tracheal Culture	N=43

No growth	31 (72.09%)	No growth	11 (25.58%)
Staphylococcus aureus (MRSA)	1 (2.32%)	Pseudomonas Aeruginosa	12 (27.90%)
Staphylococcus aureus (MSSA)	3 (6.97%)	Staphylococcus Aureus	6 (13.95%)
Psuedomonas Aeruginosa	5 (11.62%)	Klebsella Pneumonia	3 (6.97%)
Escherichia Coli	1 (2.32%)	Steptococcus species	3 (6.97%)
Streptococcus pneumonia	1 (2.32%)	Haemophilus influenza	2 (4.65%)
Burkholderia Cepacia	1 (2.32%)	Burkholderia Cepacia	2 (4.65%)

Stool for fat globules	N=43	Escherichia Coli	2 (4.65%)

Positive	21 (48.83%)	Mixed Flora	1 (2.32%)
Negative	23 (51.16%)	Candida Species	1 (2.32%)
			

*Radiology features on X-ray Chest*	*N=43*	*Radiology features on HRCT Chest*	*N=43*

Normal	1 (2.32%)	Normal	7 (16.27%)
Not performed	0	Not performed	4 (9.30%)
Consolidation	12 (27.90%)	Consolidation only	5 (11.62%)
Hyperinflation	5 (11.62%)	Consolidation with hyperinflation	4 (9.30%)
Prominent bronchovascular markings	4 (9.30%)	Consolidation with collapse	3 (6.97%)
Perihilar infiltrates	2 (4.65%)	Consolidation, collapse and hyperinflation	2 (4.65%)
Honey combing	19 (44.18%)	Bronchiectasis and hyperinflation	18 (41.86%)

MSSA=Methicillin Sensitive Staphylococcus Aureus, MRSA=Methicilin Resistant Staphylococcus Auresus, HRCT=High Resolution Computed Tomography.

## DISCUSSION

Cystic fibrosis (CF) is one of the underdiagnosed inherited diseases in developing countries. Clinical features in CF are similar to common respiratory and gastrointestinal diseases and may mimic with asthma, pneumonia and malabsorption syndromes. At the same time, high under five mortality rates in our region has overlooked CF being one of the potential differential diagnoses in such children presenting with respiratory infections and diarrhea.^8^ This results in late diagnosis and consequently increases risk of morbidity and mortality in these children. In our cohort, more than 50% patients were diagnosed after 3 years of life even though 75% patients had clinical symptoms during first year of life. This is in contrast to data reported from Europe and USA where CF patients are usually diagnosed in first year of life. El-Falaki et al showed similar results with majority of patients had symptoms in first year of life and diagnosis was made after second year of life.^9^ This delay is partly due to lack of rationalizing CF by health care provider as an important differential diagnosis and partly by non-availability of facilities assistingearly diagnosis.^10^ More than half of our cohort had parents with consanguineous marriages which is similar to Desgeorges M et al. from Lebanon who found 50% rate of consanguineous marriage in their CF population.^11^ More than 1800 mutations have been reported for CFTR gene. In our study, 12 (27.90%) patients have shown positive results for Delta F-508 genetic mutation with 5 (11.62%) were heterozygous and 7 (16.27%) were homozygous. This is consistent with Kabra et al.^8^ and Naguib et al.^12^ which shows similar results with positive delta F508 mutation seen in 19% and 25% patients respectively. Similarly, the previous data from Pakistan showed that the frequency of delta F508 mutation in Pakistani CF children is lower than reported frequency in the Caucasian population.^13^

The most common first ever clinical presentation in CF children in our population was chronic cough that was noted in 30 (69.76%) patients followed by chronic diarrhea. One patient presented only with failure to thrive in absence of associated respiratory or gastrointestinal features. Four (9.30%) patients had meconium related presentation at early neonatal age. Meconium Ileus is frequently described as a significant clinical evidence for CF and these infants should be screen promptly as advocated by Sawicka et al.^14^ from Poland and Mushtaq et al. with East London experience.^15^ Two (4.65%) patients presented with electrolyte imbalance with pseudo barter syndrome as their first CF defining feature. Metabolic alkalosis with electrolyte imbalance is one of the noteworthy clinical presentations in children with CF, especially in humid areas and timely investigations can help to establish appropriate diagnosis.^16^

Chronic productive cough was present in 39 (90.6%) patients being the most common clinical presentation in our population. About 31 (72%) patients had recurrent bronchopneumonia and 19 (44.18%) patients had chronic cough with wheezing requiring bronchodilators and inhaled steroids. Childhood wheezing in CF is frequently reported and is associated with poorer lung function at later age.^17^ Patients with CF are at augmented possibility of having nasal polyps and rhinosinusitis.^18^ In our experience, 6 (13.95%) children with CF had nasal polyp and 5 (11.62%) had chronic rhino-sinusitis. Pulmonary hypertension is associated with considerably high risk of mortality in CF patients especially with progressive pulmonary disease.^19^,^20^ Thirteen (30.23%) patients in our cohort had moderate to severe degree of pulmonary hypertension documented on two dimensional Doppler echocardiography.

Gastrointestinal manifestation in CF is related to some important mutations and its presence or absence along with its onset has been demarcated by different genetic mutations.^21^ Our study has documented 5 (11.62%) patients who had presented only with steatorrhea as the onset of CF defining symptom and around 24 (56%) patients had this presentation in the course of of their illness. These finding are similar to Kawoosa et al. who found 50% patients with diarrhea and Steatorrhea.^22^ Five (11.62%) patients have hepatobiliary-pancreatic involvement during the course of disease. Around 86% patients presented with failure to thrive in our cohort. Faltering growth in these patients is consequence of dual action of pancreatic insufficiency leading to steatorrhea and utilization of calories due to poor pulmonary health.^23^ High resolution Computed tomography (HRCT) of chest showed 18 (42%) patients with some degree of bronchiectatic changes in our patients. Certain studies advocate CT scan as a tool to detect early pulmonary deterioration and showed that serial CT scans revealed early worsening whereas serial Pulmonary Function Tests (PFTs) performed at the same time remained unaffected or declined at a gentler rate in children with CF.^24^ About 28% patients in our data showed growth of pseudomonas aeruginosa in sputum cultures followed by Staphylococcus aureus. Staphylococcus aureus is usually the first microbe seen in CF patients in early part of their life followed by Pseudomonas aeruginosa. Colonization of pseudomonas in airways at the time diagnosis indicates late recognition of condition.^25^

## CONCLUSION

Respiratory presentations predominate in CF children followed by gastrointestinal features. Nearly half of our patient had bronchiectatic changes on CT scan chest and more than quarter had pseudomonas colonization in the airways at the time of diagnosis. There is significant delay in diagnosing patient with CF resulting in early deterioration of lung function, consequently affecting their growth and nutrition. Delta F-508 mutation was found to be uncommon in our study population. Hence, it is high time that the range and dissemination of CFTR mutations in Pakistani population should be determined by completely analyzing the CFTR gene in CF patients for prompt diagnosis and early management.

### Authors Contribution

***DAA and AGB:*** Conceptualized and jointly designed the study, and drafted the initial manuscript.

***AQ and MK:*** Did the data collection, analysis and compiled the results.

***SK:*** Helped in literature review and in editing of the manuscript for final submission.
